# Stage III and oestrogen receptor negativity are associated with poor prognosis after adjuvant high-dose therapy in high-risk breast cancer

**DOI:** 10.1038/sj.bjc.6690239

**Published:** 1999-03

**Authors:** S Hohaus, L Funk, S Martin, R F Schlenk, A Abdallah, U Hahn, G Egerer, H Goldschmidt, A Schneeweiß, N Fersis, S Kaul, D Wallwiener, G Bastert, R Haas

**Affiliations:** Department of Internal Medicine V, University of Heidelberg, Heidelberg, Germany; Department of Gynaecology, University of Heidelberg, Heidelberg, Germany; Clinical Cooperation Unit, German Cancer Research Center, Molecular Haematology and Oncology

**Keywords:** breast cancer, high-dose chemotherapy, peripheral blood stem cell transplantation, prognostic indicators, tumour cells

## Abstract

We report on the efficacy and toxicity of a sequential high-dose therapy with peripheral blood stem cell (PBSC) support in 85 patients with high-risk stage II/III breast cancer. There were 71 patients with more than nine tumour-positive axillary lymph nodes. An induction therapy of two cycles of ifosfamide (total dose, 7.5 g m^−2^) and epirubicin (120 mg m^−2^) was given, and PBSC were harvested during G-CSF-supported leucocyte recovery following the second cycle. The PBSC-supported high-dose chemotherapy consisted of two cycles of ifosfamide (total dose, 12 000 mg m^−2^), carboplatin (900 mg m^−2^) and epirubicin (180 mg m^−2^). Patients were autografted with a median number of 3.7 × 10^6^ CD34+ cells kg^−1^ (range, 1.9–26.5 × 10^6^) resulting in haematological reconstitution within approximately 2 weeks following high-dose therapy. The toxicity was moderate in general, and there was no treatment-related toxic death. Twenty-one patients relapsed between 3 and 30 months following the last cycle of high-dose therapy (median, 11 months). The probability of disease-free and overall survival at 4 years were 60% and 83%, respectively. According to a multivariate analysis, patients with stage II disease had a significantly better probability of disease-free survival (74%) in comparison to patients with stage III disease (36%). The probability of disease-free survival was also significantly better for patients with oestrogen receptor-positive tumours (70%) compared to patients with receptor-negative ones (40%). Bone marrow samples collected from 52 patients after high-dose therapy were examined to evaluate the prognostic relevance of isolated tumour cells. The proportion of patients presenting with tumour cell-positive samples did not change in comparison to that observed before high-dose therapy (65% vs 71%), but a decrease in the incidence and concentration of tumour cells was observed over time after high-dose therapy. This finding was true for patients with relapse and for those in remission, which argues against a prognostic significance of isolated tumour cells in bone marrow. In conclusion, sequential high-dose chemotherapy with PBSC support can be safely administered to patients with high-risk stage II/III breast cancer. Further intensification of the therapy, including the addition of non-cross resistant drugs or immunological approaches such as the use of antibodies against HER-2/NEU, may be envisaged for patients with stage III disease and hormone receptor-negative tumours. © 1999 Cancer Research Campaign


*British Journal of Cancer* (1999) **79**(9/10), 1500–1507
© 1999 Cancer Research Campaign
Article no. bjoc.1998.0239

### Stage III and oestrogen receptor negativity are associated with poor prognosis after adjuvant high-dose therapy in high-risk breast cancer

**S Hohaus[1], L Funk[1], S Martin[1], RF Schlenk[1], A Abdallah[1], U Hahn[1], G Egerer[1], H Goldschmidt[1], A Schneeweiß[2], N Fersis[2], S Kaul[2], D Wallwiener[2], G Bastert[2] and R Haas[1,3]**

Departments of [1]Internal Medicine V and [2]Gynaecology, University of Heidelberg, Heidelberg, Germany; [3]Clinical Cooperation Unit, Molecular Haematology and Oncology, German Cancer Research Center

**Summary** We report on the efficacy and toxicity of a sequential high-dose therapy with peripheral blood stem cell (PBSC) support in 85 patients with high-risk stage II/III breast cancer. There were 71 patients with more than nine tumour-positive axillary lymph nodes. An induction therapy of two cycles of ifosfamide (total dose, 7.5 g $m^{-2}$) and epirubicin (120 mg $m^{-2}$) was given, and PBSC were harvested during G-CSF-supported leucocyte recovery following the second cycle. The PBSC-supported high-dose chemotherapy consisted of two cycles of ifosfamide (total dose, 12 000 mg $m^{-2}$), carboplatin (900 mg $m^{-2}$) and epirubicin (180 mg $m^{-2}$). Patients were autografted with a median number of $3.7 \times 10^6$ CD34+ cells $kg^{-1}$ (range, 1.9–$26.5 \times 10^6$) resulting in haematological reconstitution within approximately 2 weeks following high-dose therapy. The toxicity was moderate in general, and there was no treatment-related toxic death. Twenty-one patients relapsed between 3 and 30 months following the last cycle of high-dose therapy (median, 11 months). The probability of disease-free and overall survival at 4 years were 60% and 83%, respectively. According to a multivariate analysis, patients with stage II disease had a significantly better probability of disease-free survival (74%) in comparison to patients with stage III disease (36%). The probability of disease-free survival was also significantly better for patients with oestrogen receptor-positive tumours (70%) compared to patients with receptor-negative ones (40%). Bone marrow samples collected from 52 patients after high-dose therapy were examined to evaluate the prognostic relevance of isolated tumour cells. The proportion of patients presenting with tumour cell-positive samples did not change in comparison to that observed before high-dose therapy (65% vs 71%), but a decrease in the incidence and concentration of tumour cells was observed over time after high-dose therapy. This finding was true for patients with relapse and for those in remission, which argues against a prognostic significance of isolated tumour cells in bone marrow. In conclusion, sequential high-dose chemotherapy with PBSC support can be safely administered to patients with high-risk stage II/III breast cancer. Further intensification of the therapy, including the addition of non-cross resistant drugs or immunological approaches such as the use of antibodies against HER-2/NEU, may be envisaged for patients with stage III disease and hormone receptor-negative tumours.

**Keywords:** breast cancer; high-dose chemotherapy; peripheral blood stem cell transplantation; prognostic indicators; tumour cells

High-dose therapy with autologous stem cell support is increasingly administered to patients with high-risk breast cancer. According to a recent survey, 5886 patients with breast cancer received high-dose therapy with stem cell support in North America between 1989 and 1995 (Antman et al, 1997). The use of peripheral blood stem cells (PBSC), rather than bone marrow, and improvements in the supportive care significantly reduced the toxicity of high-dose therapy, resulting in a decrease of the 100-day mortality from 22% in 1989 to 5% in 1995. Instead of administering a single cycle of high-dose therapy, dose intensification can also be achieved by administering two or more cycles of PBSC-supported dose-escalated therapy (Dunphy et al, 1990; Ayash et al, 1994; Broun et al, 1995; Bitran et al, 1996; Haas et al, 1997). As a consequence of the substantial decrease with regard to morbidity and mortality, high-dose chemotherapy became a therapeutic option for patients with locally advanced breast cancer (Peters et al, 1993; van der Wall et al, 1995; Gianni et al, 1997; Haas et al, 1997; Somlo et al, 1997). For instance, in patients with primary breast cancer and tumour involvement of more than nine axillary lymph nodes, Gianni et al (1997) compared the efficacy of doxorubicin followed by cyclophosphamide, methotrexate and 5-fluorouracil to high-dose therapy with sequential administration of cyclophosphamide, vincristine, methotrexate, cisplatin and melphalan followed by bone marrow or PBSC transplantation. For the 67 patients treated with high-dose therapy, the probability of disease-free survival after 5 years was 57% compared with 41% for those who had received cytotoxic chemotherapy at conventional doses.

In this study, we report on 85 patients with high-risk stage II/III breast cancer, including 71 patients with more than nine tumour-positive axillary lymph nodes. Particular emphasis was put on the evaluation of prognostic factors such as stage of the disease, number of involved axillary lymph nodes, the hormone receptor status, and the presence of isolated tumour cells in samples from bone marrow obtained after high-dose therapy.

*Received 28 May 1998*
*Revised 11 September 1998*
*Accepted 25 September 1998*

*Correspondence to:* S Hohaus, Department of Internal Medicine V, University of Heidelberg, Hospitalstr. 3, 69115 Heidelberg, Germany

**Table 1** Patient characteristics

| | | | |
|---|---|---|---|
| No. of patients | 85 | | |
| Age, years | | | |
| Median | 45 | | |
| Range | 23–59 | | |
| Menopausal status | | | |
| pre- | 60 | | |
| post- | 25 | | |
| Type of surgical procedure | | | |
| Mastectomy | 57 | | |
| Breast-conserving surgery | 28 | | |
| Stage | | | |
| II A | 20 | | |
| II B | 32 | | |
| III A | 24 | | |
| III B | 9 | | |
| No. of axillary involved lymph nodes | | | |
| < 10 | 14 | | |
| 10–14 | 32 | | |
| 15–19 | 14 | | |
| > 19 | 25 | | |
| Receptor status | | | |
| ER-positive | 49 | PR-positive | 48 |
| ER-negative | 33 | PR-negative | 34 |
| n.a. | 3 | n.a. | 3 |

No., number; n.a., not available; ER, oestrogen receptor, PR, progesterone receptor.

**Table 2** Patient characteristics according to TNM stage

| | T1 | T2 | T3 | T4 |
|---|---|---|---|---|
| N1 | 20 | 32 | 10 | 8[a] |
| N2 | 4 | 6 | 4 | 1 |

[a]3 patients with inflammatory breast cancer. The lightly shadowed area ☐ denotes the 24 patients with stage III A, the darker area ☐ stage III B disease.

### PATIENTS AND METHODS

### Patients

Between July 1993 and August 1997, 85 female patients with primary breast cancer were enrolled onto this study and completed high-dose therapy by 31 December 1997. Their median age was 45 years (range, 23–59 years). According to the TNM classification, 52 patients had stage II and 33 patients had stage III disease. Fifty-seven patients underwent mastectomy, while 28 had breast-conserving surgery. The common denominator for including them into our study was the presence of high-risk factors. The majority of patients (71 of 85 patients) had tumour involvement of more than nine axillary lymph nodes, while 14 patients had a median of seven lymph nodes with additional risk factors such as hormone receptor-negative tumour, young age, high S phase, or tumour cells in the bone marrow. The patients were enrolled within 4 weeks after operation for the adjuvant treatment protocol. Patient characteristics including the number of axillary lymph nodes involved, stage of the disease and hormone receptor status are given in Tables 1 and 2. The study was conducted under the guidelines of the Joint Ethical Committee of the University of Heidelberg. Each patient gave her informed consent to participate in the study. The cut-off date of this report was 30 March 1998.

### Cytotoxic chemotherapy

Cytotoxic chemotherapy consisted of two cycles of ifosfamide, 2500 mg $m^{-2}$ as 20-h intravenous (i.v.) infusion on 3 days, and epirubicin, 40 mg $m^{-2}$ as a 4-h i.v. infusion on 3 days (Figure 1). Mesna was given at the same dose as ifosfamide on 4 days. Both cycles were supported with R-metHuG-CSF (filgrastim, 300 µg/ day$^{-1}$

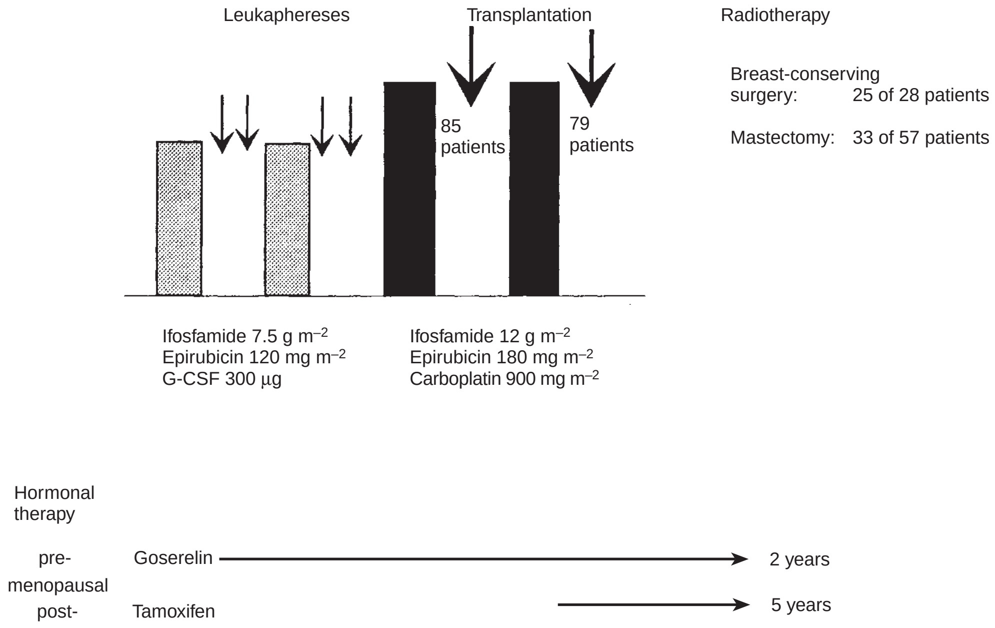

**Figure 1** Treatment plan

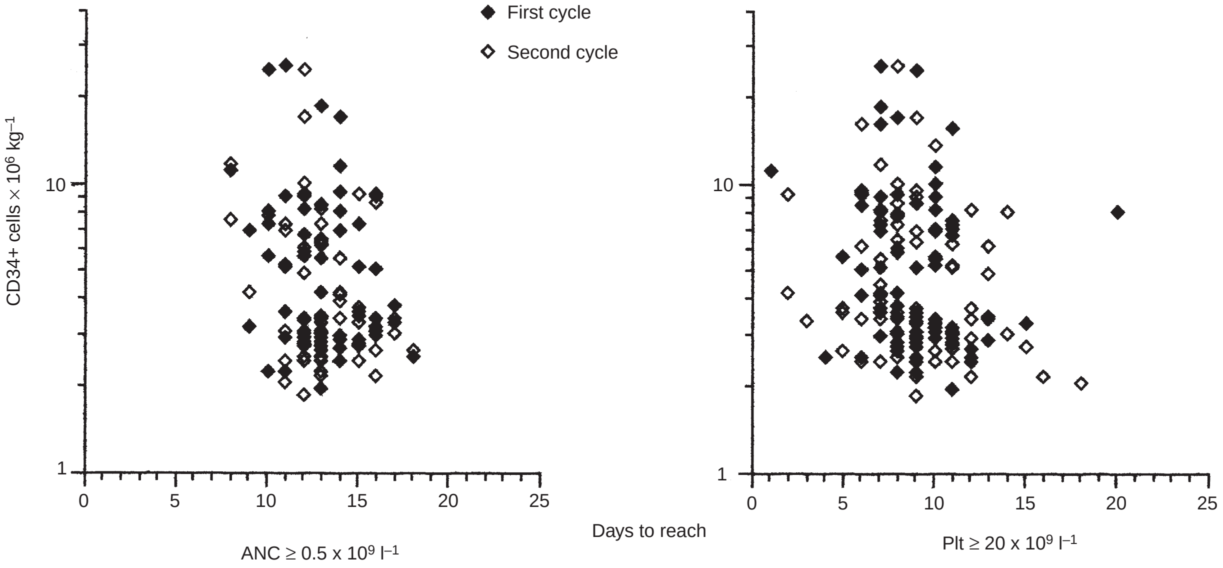

**Figure 2** Haematological reconstitution after high-dose chemotherapy supported with peripheral blood stem cells (PBSC) in relationship to the number of CD34+ cells autografted. ANC, absolute neutrophil count; Plt, platelets subcutaneously (s.c.); Neupogen®, Amgen Inc., Thousand Oaks, CA, USA). The growth factor was given to shorten the period of neutropenia and to increase the number of circulating progenitor cells during marrow recovery.

PBSC collection began when a distinct population of CD34+ cells was measurable in the peripheral blood. The leukaphereses were performed using a Fenwal CS3000 (Baxter Deutschland GmbH, Munich, Germany) or a Spectra (Cobe Laboratories, Lakewood, CA, USA). Between 10 l and 20 l were processed at flow rates of 70–150 ml $min^{-1}$. For PBSC collection, the patients received large-bore catheters in the jugular vein as previously described (Hahn et al, 1995).

The cytotoxic therapy was continued with two cycles of PBSC-supported high-dose ifosfamide (total dose of 12 000 mg $m^{-2}$), epirubicin (180 mg $m^{-2}$) and carboplatin (900 mg $m^{-2}$) (Figure 1). The dose of all drugs was delivered over a period of 5 days. Ifosfamide was given as 24-h continuous i.v. infusion, while epirubicin and carboplatin were administered over 4 and 2 h, respectively. Mesna was given at the same dose as ifosfamide, on days 1–5, followed by an additional administration of 50% of the dose on day 6.

PBSC were re-infused following 1 day after the end of cytotoxic chemotherapy, and no cytokines were given following transplantation. The patients received prophylactic antimicrobial therapy with ciprofloxacin (1000 mg $day^{-1}$) and fluconazole (400 mg $day^{-1}$). Empirical antibiotic therapy was administered for fever ≥ 38.5°C. Platelet counts above $20 \times 10^9$ $l^{-1}$ were maintained by platelet transfusions, and packed red cells were given when the haemoglobin was less than 8.0 g $dl^{-2}$.

Pre-menopausal women received anti-hormonal therapy with goserelin at a dose of 3.6 mg administered subcutaneously once per month. By starting ovarian suppression from day 0 on, we intended to suppress menstrual bleeding during the time of thrombocytopenia after high-dose therapy. Post-menopausal women received tamoxifen orally at a daily dose of 30 mg which was commenced 6 weeks following PBSC-supported high-dose therapy. The therapy with goserelin was scheduled for 2 years, while tamoxifen was envisaged for 5 years.

Twenty-five of 28 patients who underwent breast-conserving surgery received local-regional irradiation of the breast and draining lymph nodes at a dose of 50 Gy with a boost of 10 Gy to the tumour bed. For patients undergoing modified radical mastectomy, there was no general recommendation for radiotherapy of the chest wall at the beginning. From August 1996, irradiation of the chest wall and draining lymph nodes was included for all patients. As a result, 33 of 57 mastectomized patients underwent local radiotherapy.

### Immunofluorescence staining and flow cytometry

For immunofluorescence analysis, $1 \times 10^6$ mononuclear cells (MNC) of the leukapheresis product or between 20 and 50 μl of whole blood (EDTA) were incubated for 30 min at 4°C with the phycoerythrin (PE)- or fluorescein isothiocyanate (FITC)-conjugated monoclonal antibody (mAb) CD34 (HPCA-2) and CD45 (HLe-1) FITC. The mAbs were obtained from Becton-Dickinson (Heidelberg, Germany). Isotype-identical antibodies served as controls: $IgG_1$, $IgG_{2a}$ (FITC/PE-conjugated, Becton-Dickinson, Heidelberg, Germany). The cells were analysed with a Becton-Dickinson FACScan as previously described (Hohaus et al, 1993; Haas et al, 1994).

### Immunocytochemistry

Leukapheresis products and bone marrow specimens were examined for tumour cells using an immunostaining method as previously described (Haas et al, 1997; Hohaus et al, 1997). Briefly, $1 \times 10^6$ mononuclear cells were transferred onto glass slides using a cytocentrifuge (Universal-16, Hettich, Tuttlingen, Germany). Slides were fixed after air drying with formalin 3.7% (15 min) and methanol 100% (5 min). The slides were stored at –70°C in conservation medium (0.25 M saccharose, 3 mM magnesium chloride,

 © Cancer Research Campaign 1999

**Table 3** Toxicity of PBSC-supported high-dose therapy

| | Cycle 1 | Cycle 2 |
|---|---|---|
| No. of patients with | | |
| Fever ≥ 38.5°C | 76 (89%) | 60 (76%) |
| i.v. nutrition | 29 (34%) | 21 (27%) |
| Days of | | |
| Fever ≥ 38.5°C | 2 (0–12) | 2 (0–6) |
| i.v. antibiotics | 7 (0–17) | 7 (0–16) |
| i.v. nutrition | 0 (0–14) | 0 (0–14) |
| Hospital stay Post-transplantation | 14 (10–14) | 13 (9–20) |
| No. of transfusions per patient | | |
| Red blood cells | 2 (0–10) | 2 (0–6) |
| Platelets | 2 (1–18) | 2 (0–6) |

PBSC, peripheral blood stem cells; No., number; i.v., intravenous.

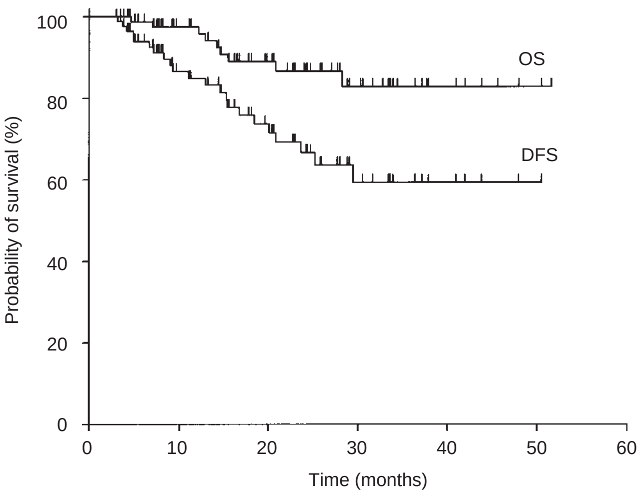

**Figure 3** Overall survival (OS) and disease-free survival (DFS) following the last cycle of adjuvant PBSC-supported high-dose chemotherapy in 85 patients with high-risk stage II/III breast cancer

50% glycerin). Immunostaining was performed using a cocktail of four monoclonal epithelial-specific antibodies. The antibody 5D3 (BioGenex, San Ramos, CA, USA) recognizes a common epitope on the intracellular cytokeratin components 8, 18 and 19, while the antibody HEA 125 (Progen Biotechnik GmbH, Heidelberg, Germany) reacts with a human surface glycoprotein specific for epithelial cells. BM7 and BM8 are murine monoclonal antibodies developed by S Kaul with a specificity for glycosylated side-chains of the breast mucin encoded by the Muc1 gene. The antibody reaction was developed with an alkaline–phosphatase anti-alkaline–phosphatase method (APAAP) (Dako, Glostrup, Denmark). Four slides containing $4 \times 10^6$ cells were examined per specimen.

### Statistical analysis

Overall survival and disease-free survival were examined as clinical outcome variables. Overall survival was defined as the time from last blood stem cell transplantation to death. Disease-free survival was defined as the time from last blood stem cell transplantation to relapse or death. Survival curves were estimated using the Kaplan–Meier product limit method (Kaplan et al, 1958). The univariate and multivariate analyses were performed to identify risk factors associated with overall and disease-free survival. Differences between the survival curves were compared using the log-rank test (Peto et al, 1972). Multivariate analysis was performed using the Cox regression model with stepwise analysis ($P$-values for entry = 0.15 and for removal = 0.05) (Cox et al, 1972). The following risk factors were first examined in a univariate analysis using the log-rank test: age, menopausal status, type of surgical procedure, stage (stage II A or B versus stage III A or B), number of tumour-positive axillary lymph nodes (group 1 < 10, group 2 10–19, group 3 > 19), histological grading (G2 versus G3), oestrogen receptor (ER) status, progesterone receptor (PR) status, radiation therapy. In a second step, variables that were significant in the univariate analysis were included in a multivariate analysis as stated above. Differences in the tumour cell concentration of bone marrow samples obtained from the same patient at different time points after high-dose therapy were tested by using the Student's paired $t$-test. The Student's $t$-test was performed using the Excel software (Version 6.0), while the other statistical computations were performed using the statistical software package SAS, Version 6.11 (SAS Institute Inc., 1995).

### RESULTS

### High-dose therapy with PBSC support

Eighty-five patients with high-risk stage II/III breast cancer were enrolled onto the study to receive PBSC-supported sequential high-dose therapy. The treatment was completed as scheduled in 79 patients, while six patients were withdrawn following the first cycle of high-dose therapy. The reasons for discontinuation were severe enterocolitis of unknown aetiology in two patients, cardiac toxicity with T-wave changes indicative for ischaemic heart disease in one patient and central nervous system (CNS) toxicity in two patients. There was one patient who declined the second cycle because of severe anxiety. The time interval between the two cycles of high-dose therapy varied between 4 and 15 weeks (median, 7 weeks). In one patient with allo-reactive antibodies against platelets, the second cycle was postponed until appropriate histocompatibility leucocyte antigen (HLA)-cross-match negative donors were found for platelet donations.

High-dose therapy was supported with a median number of $3.8 \times 10^6$ CD34+ cells $kg^{-1}$ (range, $1.9–26.5 \times 10^6$) which were mainly collected following the second cycle of granulocyte colony-stimulating factor (G-CSF)-supported induction therapy. Considering a threshold number of $2.5 \times 10^6$ CD34+ cells $kg^{-1}$ as adequate amount for sustained engraftment, the sequential high-dose therapy could be supported with portions of a single leukapheresis (LP) product in 45% of the patients, while the percentage of patients requiring between three and six LP products was only 14%.

One hundred and thirty-three LP products collected from 65 patients were assessed for the presence of tumour cells using an immunocytochemical staining method. There were only 12 patients (18%) with harvests containing tumour cells at a concentration between 0.25 and $1.25/10^6$ mononuclear cells. In contrast, 71% of bone marrow specimens obtained from 49 patients before high-dose therapy harboured tumour cells.

The time needed for haematological reconstitution following both cycles of high-dose therapy was not different. A neutrophil count of $0.5 \times 10^9\ l^{-1}$ was observed after a median time of 13 days

© Cancer Research Campaign 1999

**Table 4** Sites of relapse

| | |
|---|---|
| Local-regional | 3 |
| Contralateral breast | 2 |
| Bone | 4 |
| Lung | 1 |
| Liver | 3 |
| CNS | 3 |
| Multiple | 5 |

CNS, central nervous system.

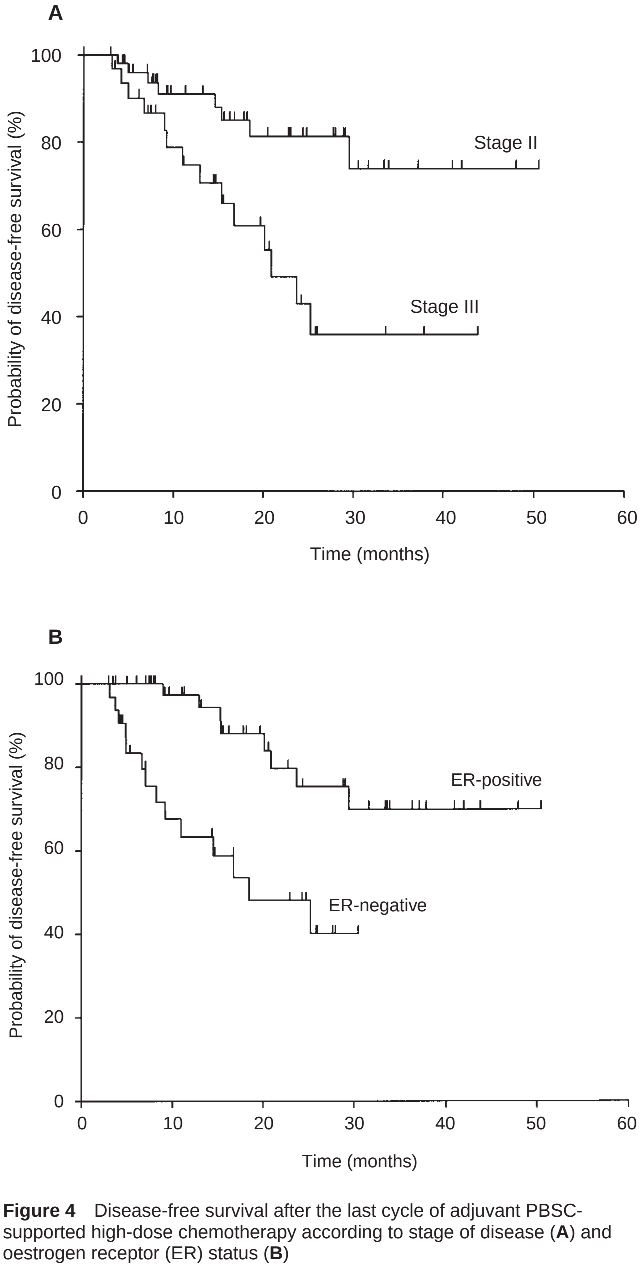

**Figure 4** Disease-free survival after the last cycle of adjuvant PBSC-supported high-dose chemotherapy according to stage of disease (**A**) and oestrogen receptor (ER) status (**B**)

(range, 8–18 days), and an unsupported platelet count of $20 \times 10^9$ $l^{-1}$ was reached after 9 days (range, 1–20) (Figure 2). The non-haematological toxicity was generally moderate. In particular, there was no day-100 treatment-related mortality. Thirty percent of the patients required total parenteral nutrition due to severe mucositis, while the median number of days with fever of ≥38.5°C following both cycles was 2 days. As a result, the number of days of i.v. antibiotic therapy amounted to a median of 7 days (Table 3).

### Therapeutic outcome

Twenty-one patients relapsed between 3 and 30 months following the last cycle of high-dose therapy (median, 11 months). As shown in Table 4, the sites of relapse were similar to those reported for patients who had received cytotoxic chemotherapy at conventional doses. Three of 27 patients who did not receive irradiation had local-regional relapse. In contrast, among 58 patients who underwent local-regional radiotherapy, there was only one patient with local relapse, which was accompanied by metastases to the lung. There were also two patients who developed tumours in the contralateral breast, after 5 and 7 months post-transplantation, respectively. As a result of surgery and radiotherapy, five of the six patients with local relapse or tumours at the contralateral site are disease-free between 1.5 and 44 months. One patient with hepatitis-C died of liver failure due to a hepatocellular carcinoma 21 months post-transplantation. The corresponding Kaplan–Meier estimate for disease-free survival at 4 years following the last cycle of high-dose therapy was 60%, while the probability of overall survival was 83% (Figure 3).

### Prognostic factor

By univariate analysis, the risk of relapse for patients with stage II disease was significantly smaller in comparison to patients with stage III disease ($P = 0.0044$). Similarly, the risk of relapse was also significantly reduced for patients with ER-positive tumours ($P = 0.0001$) when compared to patients with negative tumours. According to the multivariate analysis, the stage of disease and the ER-status were independent prognostic factors. As a consequence, patients with stage II disease had a Kaplan–Meier estimate of disease-free survival at 3 years of 74% in comparison to 36% for patients with stage III disease (Figure 4A). In the same line, the probability of disease-free survival was significantly better for patients with ER-positive tumours in comparison to those with receptor-negative ones (70% vs 40%) (Figure 4B).

### Tumour cells in bone marrow samples following PBSC

In 52 patients, bone marrow samples were collected after high-dose therapy to look for isolated tumour cells. The median time elapsed between the last cycle of high-dose therapy and the first examination was 4.5 months (range, 1–32 months) (Figure 5). In 34 of 52 patients (65%) the bone marrow samples contained tumour cells at a concentration between 0.25 and 33 cells/$10^6$ mononuclear cells (median, 2 cells/$10^6$).

The concentration of tumour cells in bone marrow specimens decreased in the course of time which had elapsed since the end of high-dose therapy (Figure 5A). An intraindividual examination including 25 patients from whom bone marrow samples were obtained on at least two occasions, showed a significant decrease of the tumour cell concentration in 16 patients and disappearance below detection limit in five patients ($P < 0.05$) (Figure 5B). Four patients had repeatedly negative samples, while there were also five patients presenting with a median increase of 0.5 tumour cells/$10^6$ MNC.

*© Cancer Research Campaign 1999*

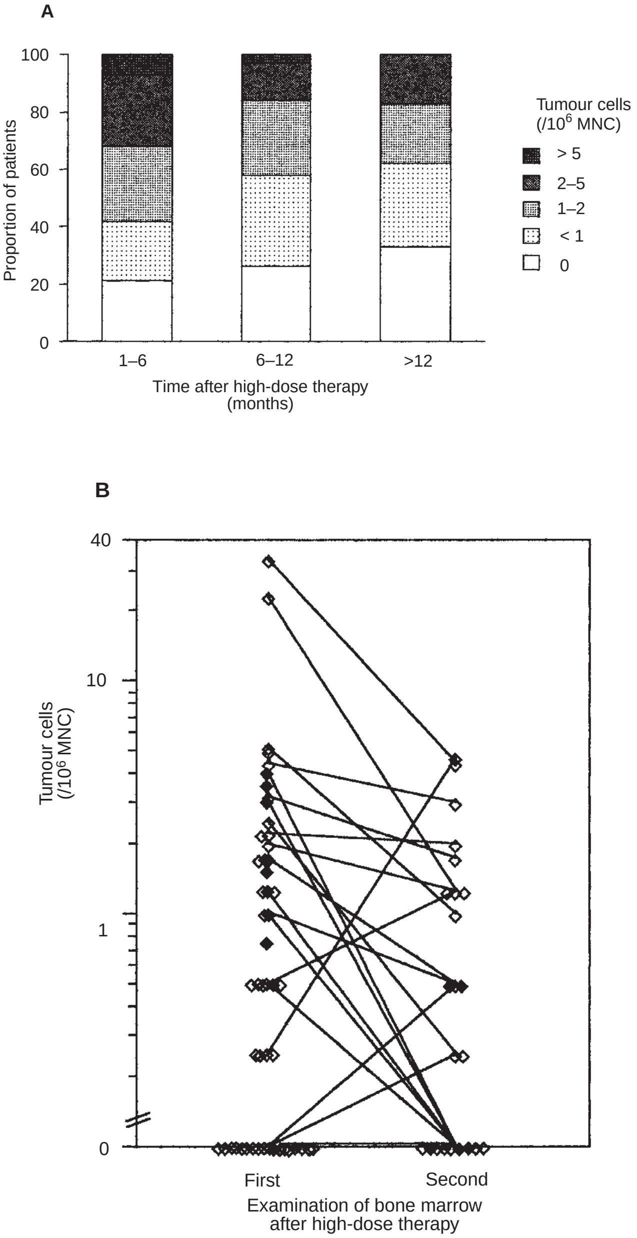

**Figure 5** Detection of tumour cells in bone marrow samples obtained after the last cycle of PBSC-supported high-dose chemotherapy. Patients who are disease-free are symbolized by open diamonds and patients with relapse by closed diamonds. (**A**) The proportion of patients with negative or small numbers of tumour cells increases during the course of time. (**B**) Concentration of tumour cells at first (52 patients) and second (25 patients) examination after high-dose therapy. In 16 of the 25 patients permitting an intra-individual comparison, a decrease in the concentration of the tumour cells could be observed. Four patients had repeatedly negative samples, while five patients showed an increase

It was interesting to note that the concentration of tumour cells and their changes over time were not different between the patients who relapsed and those who were disease-free (Figure 5B). In particular, patients with relapse had a median number of 1.5 tumour cells/$10^6$ MNC, and in four of five patients even a decrease in the concentration of tumour cells could be observed on the occasion of a second follow-up visit.

### DISCUSSION

Following conventional cytotoxic chemotherapy, long-term disease-free survival is only achieved in 15–30% of the patients with primary breast cancer and tumour involvement of more than nine axillary lymph nodes (Valagussa et al, 1978; Fisher et al, 1984). As a consequence, high-dose therapy has been envisaged to improve the treatment results for this particular group of patients. Dose escalation in the adjuvant setting appears to be acceptable as a result of better supportive care which also includes the use of PBSC rather than bone marrow for autografting (Antman et al, 1997; Haas et al, 1997). The toxicity data of our study support this notion. Following a total of 164 cycles of high-dose therapy with ifosfamide, epirubicin and carboplatin administered to 85 patients, there was no treatment-related toxic death, while the treatment had to be discontinued after the first cycle of high-dose therapy in only 7% of the patients. As a result of the relatively moderate toxicity, the high-dose therapy will be administered in an outpatient setting for patients with a care giver.

The treatment resulted in a probability of disease-free survival of 60% at 4 years following the last cycle of high-dose therapy. The data are similar to those reported by other groups and suggest that high-dose therapy may be superior to cytotoxic chemotherapy at conventional doses (Gianni et al, 1997). Still, a significant proportion of patients treated with high-dose therapy is at risk for relapse and these patients eventually die of progressive disease. We therefore looked for prognostic factors associated with an increased risk of relapse. By multivariate analysis, stage III disease and ER-negativity were independent prognostic factors associated with an increased risk of relapse. This finding is in line with data of Somlo et al (1997) showing that patients with stage III B disease had a greater likelihood of relapse compared to patients with stage II disease. Similar to our data, the lack of hormone receptor expression was also associated with bad prognosis. Since the presence of oestrogen receptors is also associated with response to anti-hormonal treatment, the better disease-free survival observed in patients with ER-positive tumours may also reflect the efficacy of the anti-hormonal treatment that we administered post-transplantation. Gianni et al (1997) observed a better disease-free survival in patients presenting with 10–20 tumour-positive axillary lymph nodes in comparison to patients with more than 20 lymph nodes, a finding that we could not confirm in our patient cohort.

The relevance of radiotherapy has also to be addressed. We observed local-regional relapses in three of 24 patients who underwent mastectomy without receiving radiotherapy thereafter. In contrast, among 58 patients with local-regional irradiation, there was only one patient presenting with a local relapse and lung metastases. These data suggest that patients with locally advanced breast cancer are still at risk for local relapse despite the high-dose therapy. Apparently they benefit from involved-field radiotherapy which is accompanied by moderate toxicity (Marks et al, 1992; van der Wall et al, 1996).

In principle, relapses following PBSC-supported adjuvant therapy could result from reinfusion of tumour cells contained within the autograft or from resistant tumour cells in vivo. Isolated tumour cells in leukapheresis products were found in 18% of the patients. Still, the proportion of patients who developed a relapse following autografting with tumour cell-positive harvests was similar to the relapse rate in patients who received an autograft free of tumour cells. In any case, before high-dose therapy the incidence

© Cancer Research Campaign 1999

of positive samples and the concentration of tumour cells were smaller in leukapheresis products than in bone marrow specimens. This finding is in line with data reported by Ross et al (1993).

We also looked for tumour cells in bone marrow samples obtained after high-dose therapy to evaluate the prognostic significance of isolated tumour cells. It was remarkable that 65% of the patients harboured tumour cells in the bone marrow following PBSC-supported high-dose therapy. Still, a significant decrease in the concentration of tumour cells was observed on the occasion of further follow-up visits. The prognostic relevance of our finding is not clear at the moment, as the median concentration of tumour cells in the bone marrow at the first examination and the reduction noted at follow-up visits were not different between patients with relapse and those who were disease-free. This finding suggests that isolated tumour cells in the bone marrow may not reflect the fate of disseminated tumour cells in other organs such as lung or liver. These are preferred sites for metastases, suggesting that the bone marrow may not provide the appropriate micro-environment for the growth of epithelial tumour cells. This is different from the malignant cells in patients with haematological malignancies. For instance, patients with chronic myeloid leukaemia who have low, decreasing or undetectable levels of BCR-ABL RNA expression have a reduced risk of relapse, while patients with increasing levels of BCR-ABL RNA are at increased risk (Lin et al, 1996). Similarly, persistence of PCR-positive cells after high-dose therapy in patients with t(14;18)-positive follicular lymphoma is also associated with a greater likelihood of relapse (Gribben et al, 1993).

In conclusion, patients with stage III disease and with ER-negative tumours have a poor prognosis despite sequential high-dose therapy. Further intensification of the therapy by administering additional cycles of high-dose therapy with non-cross-resistant cytotoxic drugs is feasible, as we have shown for patients with metastatic disease (Hohaus et al, 1998). Immunological treatment modalities including the use of antibodies against antigens expressed on the surface of breast cancer cells such as HER-2/NEU, breast mucins or the epithelial glycoprotein 17-1A might also be envisaged in conjunction with the high-dose therapy.

### ACKNOWLEDGEMENTS

We thank the nursing staff of the Department of Internal Medicine V for their outstanding care of the patients. We are grateful to Petra Schmidt, Margit Pförsich, Miriam Weis, Magdalena Volk, Kirsten Flentje and Evi Holdermann for excellent technical assistance. We thank Ulla Scheidler and Brigitte Saur for expert secretarial help.

### REFERENCES

Antman KH, Rowlings PA, Vaughan WP, Pelz CJ, Fay JW, Fields KK, Freytes CO, Gale RP, Hillner BE, Holland HK, Kennedy MJ, Klein JP, Lazarus HM, McCarthy Jr PL, Saez R, Spitzer G, Stadtmauer EA, Williams SF, Wolff S, Sobocinski KA, Armitage JO and Horowitz MM (1997) High-dose chemotherapy with autologous hematopoietic stem-cell support for breast cancer in North America. *J Clin Oncol* **15**: 1870–1879

Ayash LJ, Elias A, Wheeler C, Reich E, Schwartz G, Mazanet R, Tepler I, Warren D, Lynch C, Gonin R, Schnipper L, Frei E and Antman K (1994) Double dose-intensive chemotherapy with autologous marrow and peripheral blood progenitor cell support for metastatic breast cancer: a feasibility study. *J Clin Oncol* **12**: 37–44

Bitran JD, Samuels B, Klein L, Hanauer S, Johnson L, Martinec J, Harris E, Kempler J and White W (1996) Tandem high-dose chemotherapy supported by hematopoietic progenitor cells yield prolonged survival in stage IV breast cancer. *Bone Marrow Transplant* **17**: 157–162

Broun ER, Sridhara R, Sledge GW, Loesch D, Kneebone PH, Hanna M, Hromas R, Cornetta K and Einhorn LH (1995) Tandem autotransplantation for the treatment of metastatic breast cancer. *J Clin Oncol* **13**: 2050–2055

Cox DR (1972) Regression models and life tables. *J R Stat Soc (B)* **34**: 187–220

Dunphy FR, Spitzer G, Buzdar AU, Hortobagyi GN, Horwitz LJ, Yau JC, Spinolo JA Jagannath S, Holmes F, Wallerstein RO, Bohannan PA and Dicke KA (1990) Treatment of estrogen receptor negative or hormonally refractory breast cancer with double high-dose chemotherapy for intensification and bone marrow support. *J Clin Oncol* **8**: 1207–1216

Fisher E, Sass R and Fisher B (1984) Pathologic findings from the national surgical adjuvant project for breast cancers (protocol no. 4). *Cancer* **53**: 712–723

Gianni AM, Siena S, Bregni SM, Di Nicola M, Orefice S, Cusumano F, Salvadori B, Luini A, Greco M, Zucali R, Rilke F, Zambetti M, Valagussa P and Bonadonna G (1997) Efficacy, toxicity, and applicability of high-dose sequential chemotherapy as adjuvant treatment in operable breast cancer with 10 or more involved axillary nodes: five-year results. *J Clin Oncol* **15**: 2312–2321

Gribben JG, Neuberg D, Freedman AS, Gimmi CD, Pesek KW, Barber M, Saporito L, Woo SD, Coral F, Spector N, Rabinowe SN, Grossbard ML, Ritz J and Nadler LM (1993) Detection by polymerase chain reaction of residual cells with the bcl-2 translocation is associated with increased risk of relapse after autologous bone marrow transplantation for B-cell lymphoma. *Blood* **81**: 3449–3457

Haas R, Möhle R, Frühauf S, Goldschmidt H, Witt B, Flentje M, Wannenmacher M and Hunstein W (1994) Patient characteristics associated with successful mobilizing and autografting of peripheral blood progenitor cells in malignant lymphoma. *Blood* **83**: 3787–3794

Haas R, Schmid H, Hahn U, Hohaus S, Goldschmidt H, Murea S, Kaufmann M, Wannenmacher M, Wallwiener D, Bastert G and Hunstein W (1997) Tandem high-dose therapy with ifosfamide, epirubicin, carboplatin and peripheral blood stem cell support is an effective adjuvant treatment for high-risk primary breast cancer. *Eur J Cancer* **33**: 372–378

Hahn U, Goldschmidt H, Salwender H, Haas R and Hunstein W (1995) Large-bore central venous catheters for the collection of peripheral blood stem cells. *J Clin Apheresis* **10**: 12–16

Hohaus S, Goldschmidt H, Ehrhardt R and Haas R (1993) Successful autografting following myeloablative conditioning therapy with blood stem cells mobilized by chemotherapy plus rhG-CSF. *Exp Hematol* **21**: 508–514

Hohaus S, Pfoersich M, Murea S, Abdallah A, Lin YS, Funk L, Voso MT, Kaul S, Schmid H, Wallwiener D and Haas R (1997) Immunomagnetic selection of CD34+ peripheral blood stem cells for autografting in patients with breast cancer. *Br J Haematol* **97**: 881–888

Hohaus S, Wallwiener D, Martin S, Voso MT, Huober J, Fersis N, Bastert G and Haas R (1998) Efficacy and toxicity of sequential high-dose therapy with peripheral blood stem cell support in patients with high-risk breast cancer. *Semin Oncol* **25**: 7–11

Kaplan EL and Meier P (1958) Non-parametric estimation from incomplete observation. *J Am Stat Assoc* **47**: 457–481

Lin F, van Rhee F, Goldman JM and Gross NC (1996) Kinetics of increasing BCR-ABL transcript numbers in chronic myeloid leukemia patients who relapse after bone marrow transplantation. *Blood* **87**: 4473–4478

Marks C, Halperin E, Prosnitz L, Ross M, Vredenburgh JJ, Rosner GL and Peters W (1992) Post-mastectomy radiotherapy following adjuvant chemotherapy and autologous bone marrow transplantation for breast cancer patients with greater than or equal to 10 positive axillary lymph nodes. Cancer and Leukemia Group B. *Int J Rad Oncol Biol Phys* **23**: 1021–1026

Peters WP, Ross M, Vredenburgh JJ, Meisenberg B, Marks LB, Winer E, Kurtzberg J, Bast RC Jr, Jones R, Shpall EJ, Wu K, Rosner G, Gilbert C, Mathias B, Consighio D, Pehosw, Henderson IC, Norotn L, Weiss R, Budman D and Huid D (1993) High-dose chemotherapy and autologous bone marrow support as consolidation after standard-dose adjuvant therapy for high-risk primary breast cancer. *J Clin Oncol* **11**: 1132–1143

Peto R and Peto J (1972) Asymptotically efficient rank invariant test procedures. *J R Stat Soc (A)* **135**: 185 ff

Ross AA, Cooper BW, Lazarus HM, Mackay W, Moss TJ, Ciobanu N, Tallman MS, Kennedy MJ, Davidson NE, Sweet D, Winter C, Jansen J, Akard L, Copeland E, Kohn DJ and Werner NE (1993) Detection and viability of tumour cells in peripheral blood stem cell collections from breast cancer patients using immunocytochemical and clonogenic assay techniques. *Blood* **82**: 2605–2610

Somlo G, Doroshow JH, Forman SJ, Odom-Maryon T, Lee J, Chow W, Hamasaki V, Leong L, Morgan Jr, R, Margolin K, Raschko J, Shibata S, Tetef M, Yen Y, Simpson J and Molina A (1997) High-dose chemotherapy and stem-cell rescue in the treatment of high-risk breast cancer: prognostic indicators of progression-free and overall survival. *J Clin Oncol* **15**: 2882–2893

*© Cancer Research Campaign 1999*

Valagussa P, Bonadonna G and Veronesi U (1978) Patterns of relapse and survival following radical mastectomy: analysis of 716 consecutive patients. *Cancer* **41**: 1170–1178

van der Wall E, Mooijen WJ, Baars JW, Holtkamp MJ, Schorangel JH, Richel DJ, Rutgers EJ, Slaper-Cortenbach IC, van der Schoot CE and Rodenhuis S (1995) High-dose carboplatin, thiotepa and cyclophosphamide (CTC) with peripheral blood stem cell support in the adjuvant therapy of high-risk breast cancer: a practical approach. *Br J Cancer* **71**: 857–862

van der Wall E, Schaake-Koning CC, van Zandwijk N, Baars JW, Schornagel J, Richel DJ, Rutgers EJ, Borger JH, Beijnen JH and Rodenhuis S (1996) The toxicity of radiotherapy following high-dose chemotherapy with peripheral blood stem cell support in high-risk breast cancer: a preliminary analysis. *Eur J Cancer* **32A**: 1490–1497

© Cancer Research Campaign 1999
